# Supplementary material for: Shikimate Kinase Plays Important Roles in Anthocyanin Synthesis in Petunia
Source: Int J Mol Sci. 2022 Dec 15;23(24):15964. doi: 10.3390/ijms232415964 (PMC9786173; doi:10.3390/ijms232415964)
Supplement: Supplementary file 1 [file ijms-23-15964-s001.zip › Supplemental.pdf]

Title: Shikimate kinase plays important roles in anthocyanin synthesis in petunia

Table S1. Primer sequences of *PhSK* used in subcellular localization

Gene	Forward primer (5′→3′)	Reverse primer (5′→3′)
PhSK	TCACCATTTACGAACGATAGCCATGGC AATGGAGGCTAGAGTTTCACA	AGCTCCTCGCCCTTGCTCACCATGGAGA CCTCTAGAGTAATTTCAG

Table S2. Primer sequences of used in quantitative real-time PCR

Gene	Forward primer (5′→3′)	Reverse primer (5′→3′)
PhSK	TATTTACTTTCTTGACGAGGAG	TTGCTGGATGTTCATTCTTA
PhDHQ/SDH	ACCAAGGGTTGATGATAC	CCCGCAAGAGTCTAGTGA
PhEPSPS1	GGAGCAACCGTTGAAGAAGG	AGCAGCAAGAGAAAAAGCCA
PhCHSA	TTCAGCAGCCCAAACTCT	CCCACCTGGATGAGCAAT
PhF3'5'H	GTTGTAGTGGCGGAGATG	ATGTTGAAATACCCTGCT
PhCYP	AGGCTCATCATTCCACCGTGT	TCATCTGCGAACTTAGCACCG

Table S3. Primer sequences used in VIGS

Name	Forward primer (5′→3′)	Reverse primer (5′→3′)
pTRV2-PhSK	CGCGGATCCAGGGTATTAGTGTTTG GTTA	CCGGAATTCTAGAGTAATTTCAGGA GGAG
pTRV2-GFP	ACGCGTGAGCTCGGTACCGGAT CCGATCTCGAGTGGAGGCATTCC AACATTG	TCTGTGAGTAAGGTTACCGAAT TCCCAGAGCTCATTCAAGACCT TCACCAG
pTRV1	AACCTGGGCGAAGGACACAC	GGACTCAGATGCCGAATACA
pTRV2	TATTATTACGGACGAGTGGAC	ACCTAAAACTTCAGACACGGA

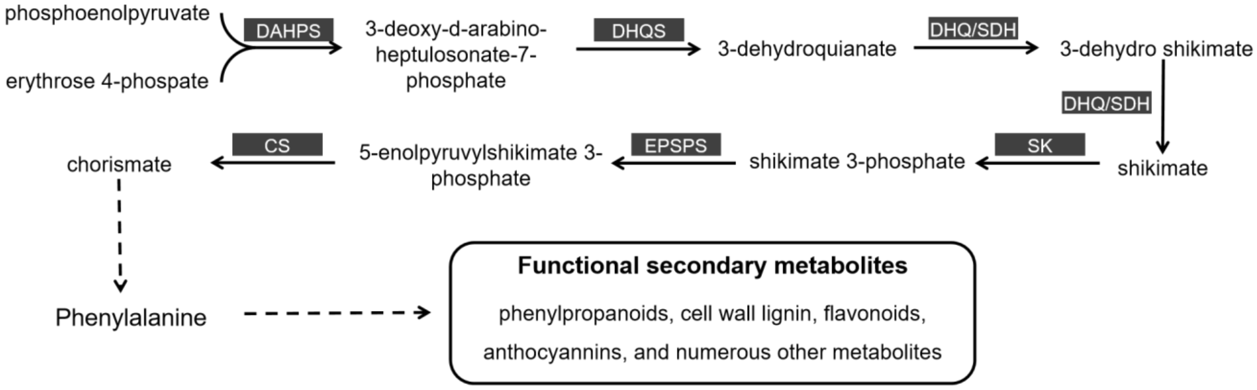

Figure S1. The shikimate biosynthesis in plants. Shikimate biosynthesis starting from phosphoenolpyruvate (PEP) and D-erythrose 4-phosphate is described with characterized enzymes and reported intermediate metabolites. The dotted lines indicate more than one reaction, and the solid lines indicate only one step in the reaction. The black boxes indicate the enzymes. Abbreviation: DAHPS, 3-deoxy-D-arabino-heptulosonate 7-phosphate synthase; DHQS, 3-dehydroquinate synthase; DHQ/SDH, 3-dehydroquinate dehydratase; SK, shikimate kinase; EPSPS, 3-phosphoshikimate 1-carboxyvinyltransferase; CS, chorismate synthase.

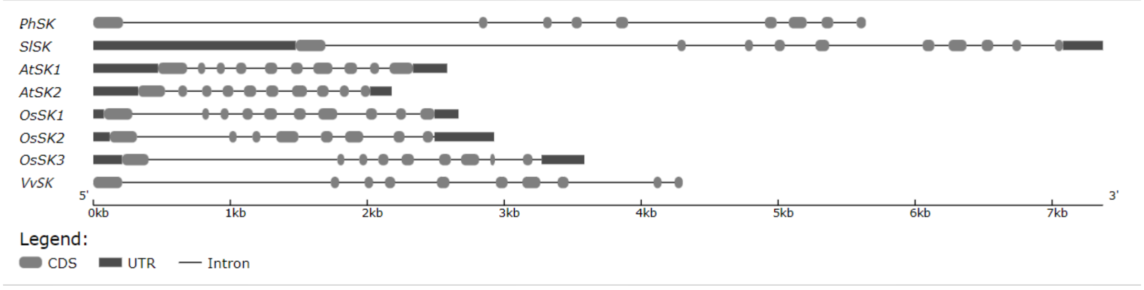

Figure S2. Gene structures of *SK*s. Names of genes are given in uppercase letters, and their origin is indicated by a two-letter prefix. Gene structure of *Petunia hybrida PhSK* (Peaxi162Scf00359g00118.1), *Solanum lycopersicum SlSK* (Solyc04g051860.3.1), *Arabidopsis thaliana AtSK1* (NM_201778.3), *AtSK2* (NM_201778.3), *Oryza sativa OsSK1* (NM_001401978.1), *OsSK2* (XP_015641676.1), *OsSK3* (XP_015636368.1), *Vitis vinifera VvSK* (NM_001281087.1).

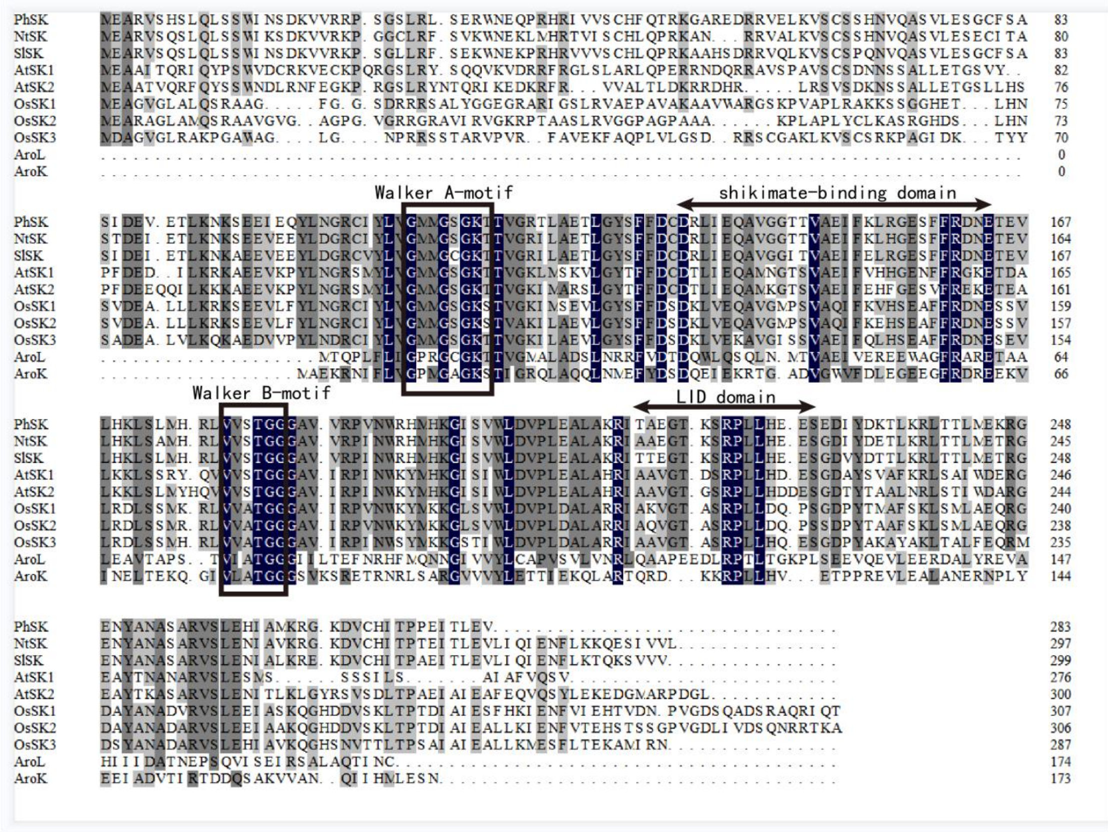

Figure S3. Sequence alignment of SK. Predicted amino acid sequence alignments of PhSK with *Nicotiana tabacum* NtSK (NP_001312965.1), *Solanum lycopersicum* SlSK (NP_001234112.1), *Arabidopsis thaliana* AtSK1 (NP_001077936.1), AtSK2 (NP_195664.2), *Oryza sativa* OsSK1 (XP_015626759.1), OsSK2 (XP_015641676.1), OsSK3 (XP_015636368.1), *Escherichia coli* AroL (WP_000193393.1), AroK (WP_000818618.1). Identical amino acids are shaded in black and conserved changes are in grey. The deep grey shading represents identical residues in eight of ten sequences, and the light grey shading indicates six similar residues of ten sequences. The black boxes show the conserved Walker A-motif and Walker B-motif respectively. The black arrows represent the shikimate-binding domain and LID domain.

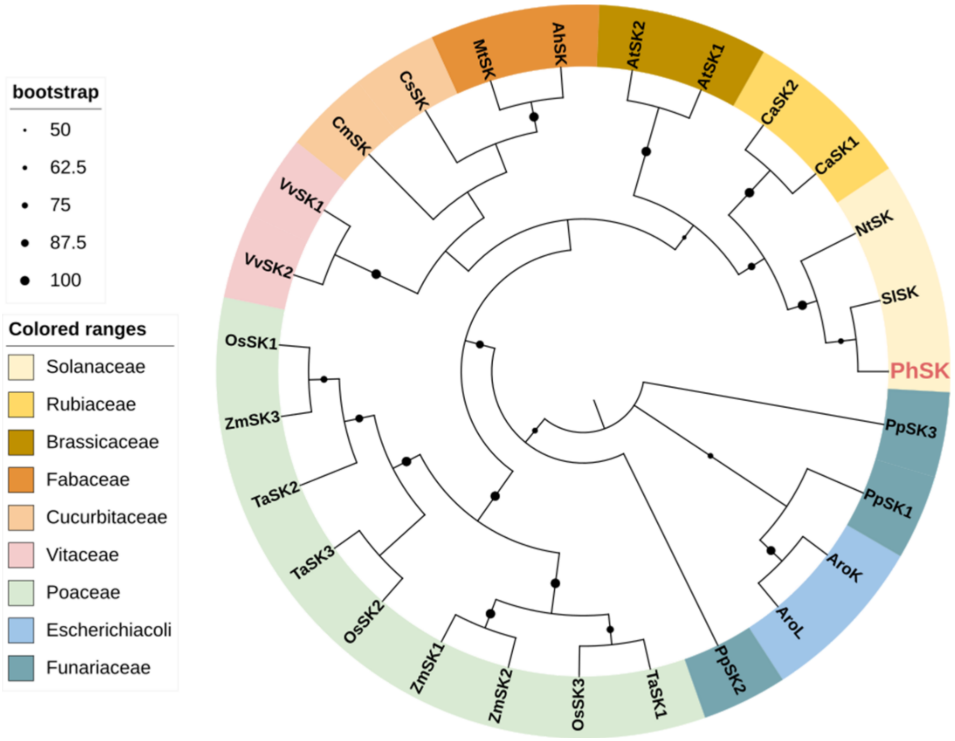

Figure S4. Phylogenetic tree displaying the similarities of SKs among different species by Maximum Likelihood method. The dendrogram was constructed using TBtools software and visualized by iTOL, an online tool. Names of proteins are given in uppercase letters, and their origin is indicated by a two-letter prefix. Phylogenetic tree of PhSK, *Nicotiana tabacum* NtSK (NP_001312965.1), *Solanum lycopersicum* SlSK (NP_001234112.1), *Arabidopsis thaliana* AtSK1 (NP_001077936.1), AtSK2 (NP_195664.2), *Oryza sativa* OsSK1 (XP_015626759.1), OsSK2 (XP_015641676.1), OsSK3 (XP_015636368.1), *Escherichia coli* AroL (WP_000193393.1), AroK (WP_000818618.1), *Medicago truncatula* MtSK(XP_039684838.1), *Vitis vinifera* VvSK1 (NP_001268016.1), VvSK2 (ACY29658.1), *Cucumis melo var. makuwa* CmSK(TYK12958.1), *Cucumis sativus* CsSK (NP_001292691.1), *Physcomitrium patens* PpSK1 (XP_024392957.1), PpSK2(XP_024377384.1), PpSK3 (XP_024387006.1), *Triticum aestivum* TaSK1 (XP_044414081.1), TaSK2 (XP_044426537.1), TaSK3 (XP_044326614.1), *Zea mays* ZmSK1 (NP_001359309.1), ZmSK2 (ACG38590.1), ZmSK3 (NP_001130232.1), *Arachis hypogaea* AhSK (QHO58581.1), *Coffea arabica* CaSK1 (XP_027077571.1), CaSK2 (XP_027077574.1).

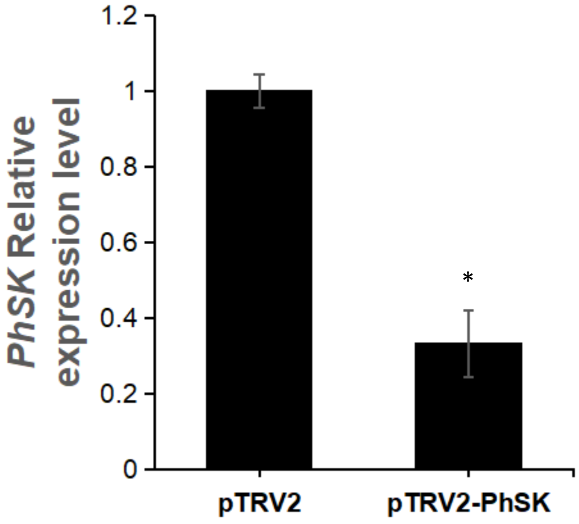

Figure S5. Effects of pTRV2-PhSK treatment on the expression of *PhSK* in corollas. *Cyclophilin* (*CYP*, accession no. EST883944) was used as the internal reference gene for the quantification of cDNA abundance. The data are presented as the means ± SDs (n = 3). The statistical analysis was performed using the one-way analysis of variance (ANOVA) followed by Duncan's multiple range test (DMRT) with three biological replicates. P values ≤0.05 were considered significant.

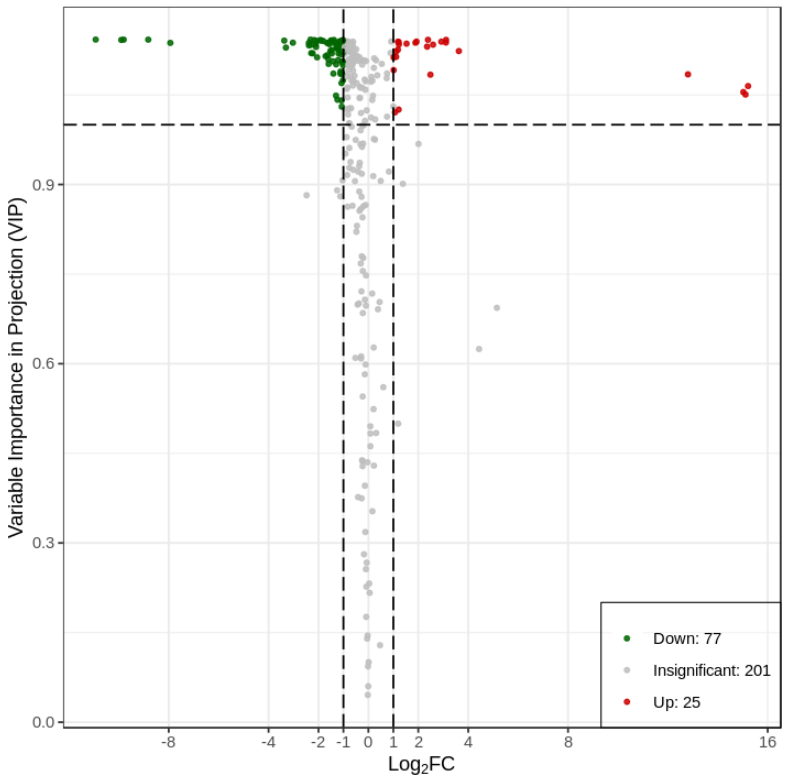

Figure S6. Volcano plot of the differential metabolites in *PhSK*-silenced compared with control petunia corollas. The green dots in the figure represent the differentially expressed metabolites that were decreased, the red dots represent the differentially expressed metabolites that were increased, and the grey color indicates metabolites that were not significantly different.

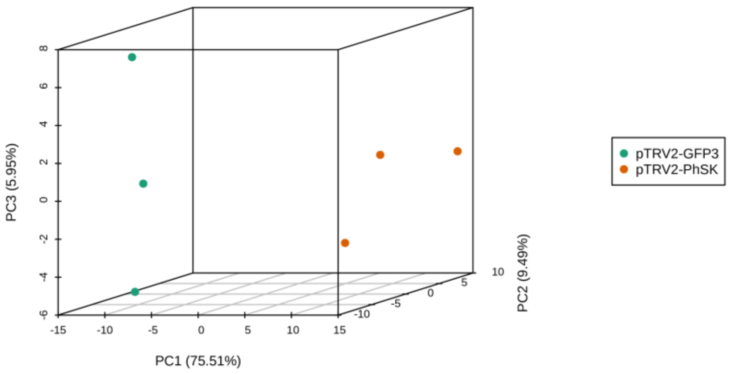

Figure S7. PCA plot of the metabolome of *PhSK*-silenced and control petunia corollas.
